# What Does the Future Hold for a Surgical Trainee? This Lockdown Is Not a Letdown Yet: A Survey on Moodle Learning Management System as a Part of Blended Learning During COVID-19 Pandemic

**DOI:** 10.7759/cureus.16690

**Published:** 2021-07-28

**Authors:** Shehzadi Rimsha, Foad Ali Moosa, Farhan Zaheer, Mohammed Taha Kamal, Aamina Majid

**Affiliations:** 1 General Surgery, Civil Hospital Karachi/Dow University of Health Sciences, Karachi, PAK

**Keywords:** moodle, pakistan, e-learning, blended learning, general surgery, covid-19, learning management system, education

## Abstract

Background

The COVID-19 pandemic brought about a major shift in the educational training of surgical trainees. As the Lockdown was implemented and the daily workforce reduced, an alternate method was employed to provide uninterrupted learning. Blended learning that includes virtual learning with face-to-face learning/teaching was utilized for the surgical trainees. MOODLE (Modular object-oriented dynamic learning environment), an open-source learning management system, was integrated as an Online Component of our Blended Learning Program. We aimed to evaluate the perception of postgraduate trainees of General Surgery regarding the benefits and limitations of Blended Learning, particularly its online component, i.e., Moodle LMS, for the betterment of surgical -education during the COVID-19 pandemic.

Material and Methods

Thirty-three postgraduate general surgery trainees were enrolled in a blended learning program, in which its online component, Moodle LMS, comprised four major topics on General Surgery. A questionnaire was provided to the trainees to obtain feedback on blended learning in general, and Moodle LMS was mainly themed on the Likert scale.

Results

The approach of blended learning was positively received by the participants, the majority of whom were females (75%) and comprising of Year 1 residents (33.3%). Nearly half of the participants found Moodle LMS user-friendly, practical and a good platform for learning. However, nearly two thirds (60.6%) were uncertain if it ever helped in applying knowledge to interpret laboratory and radiological results for patient management. Even then, most of them found that the face-to-face component of blended learning helped them develop specific clinical and surgical skills (42.4%). Emphatically, 78.7% would recommend it for surgical training.

Conclusion

Blended learning was found to be beneficial in the training process of surgical postgraduates in the current COVID-19 pandemic situation. We recommend it for the training of doctors for optimized learning.

## Introduction

The current COVID-19 pandemic crisis has affected almost every aspect of life around the globe. One area which has been drastically affected by this pandemic is education in medical colleges and postgraduate training institutes. Almost all the medical colleges and institutions across Pakistan have been affected by repeated partial or complete shutdowns to curtail the rapid spread of the COVID-19 pandemic and ensure students and the staff [1,2].

The current situation led to a massive challenge for educators to develop an alternate effective distant e-learning system to provide and sustain quality medical education to the learners. This has provided an opportunity for them to utilize online teaching and learning tools to fill in the gap created by the disruption of traditional teaching due to the COVID-19 pandemic [2]. Given the United Nations Educational, Scientific and Cultural Organization’s (UNESCO) suggestion for educational institutes to adopt Virtual Teaching in place of traditional teaching, medical educators have started relying on virtual classrooms and open-source software, which can facilitate in providing online courses and engage students with their educators simultaneously [3].

This software is the ‘Learning Management System’ or LMS, defined as a web-based application with integrated software, providing an online learning environment programmed to handle interactive online courses. LMS provides an online platform for student-teacher interaction (synchronous and asynchronous), conducting assessments, keeping a record of reports, learning progress, student activities, and providing learners’ feedback [4,5]. Few examples of LMS used in educational institutions include Moodle, Blackboard, and Desire2Learn [6,7]. However, online clinical teaching for postgraduate surgical trainees presents a difficult challenge for educators. As per the World Health Organization (WHO) guidelines, hospitals worldwide had to postpone elective surgeries as a preventive measure against the spread of COVID-19 and for patient safety. The face-to-face teaching activities of postgraduates, including ward rounds, elective theatres, case-based learning, journal club, post-emergency morbidity and mortality meetings, all have been interrupted globally [8].

Conducting surgical postgraduate teaching online has its limitations because work-based clinical learning requires active interaction with the patient, and real-time experience of surgical procedures is needed for an active learning experience. Hence a blended mode of learning (face to face plus online) was developed to overcome the lack of hands-on training and continuing medical education online. Blended learning (BL) combines the merits of both online and face-to-face learning, creating significant collaboration between students, educators, and patients [9].

Moodle LMS is popular amongst many educational organizations worldwide, aiming to incorporate e-Learning solutions in their institutions due to the lack of required infrastructure and resources. Moodle being an open-source LMS, aims to provide a secure and integrated system to create individualized learning environments. It is easy to use, customizable as per the need for large or small learning utilities, provides a single platform to users to have online courses, and makes personalized blogs, wikis, and forums [10,11]. Teachers find it interesting to transfer and share knowledge, conduct exercises and tests as formative assessments compared to conventional lecture-based teaching [12].

On literature search, not many studies have been conducted to evaluate the effectiveness of Blended teaching platforms for postgraduate surgical trainees.

Our objective was to identify the perception of postgraduate trainees of General Surgery regarding the benefits and limitations of Blended Learning, particularly its online component, i.e., Moodle LMS. The conclusion drawn from this study will help the surgical trainees and their teachers to find an alternate platform for conventional teaching and to cope up with the academic loss during the COVID-19 pandemic.

## Materials and methods

Blended Learning

**Figure 1:**
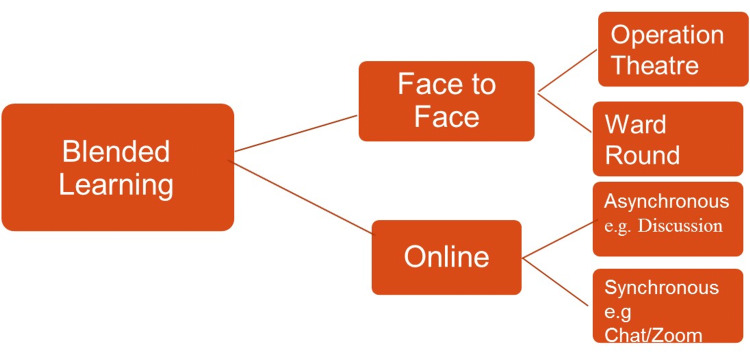
The components of blended learning

Blended learning can be explained in figure 1

The online component of the blended learning

Moodle LMS

The online component of the teaching course was set up on Moodle LMS titled “Blended Teaching & Learning Course for Postgraduate Surgical trainees, Surgical Unit, CHK”. Course layout and learning objectives were outlined on four major topics, and four weeks were allotted to each. The topics were as follows; ABC & Management of Major Trauma (ATLS Guidelines), Types & Management of Abdominal wall defects including Hernias, Diagnosis & Management of Acute Abdomen and Clinical Examination and Surgical Skills.

Online Meeting Forum

By far, the most generously accessed online tool during the COVID-19 pandemic has been for Virtual Meetings & conferences. The Zoom Meeting application was incorporated with Moodle LMS program to conduct twice-weekly meetings, comprised of post-emergency meetings and topic presentations on assigned topics by the teachers. These PowerPoint presentations were immediately uploaded on Moodle LMS after the Zoom meetings and were available for students for viewing and downloading.

Features and Built-In Tools of Moodle LMS

Multiple built-in tools available in Moodle LMS were utilized in our learning course. Files were used for uploading teaching material and PowerPoint presentations; Forum for asynchronous discussion; URL to provide External URL Links, addresses for reference material, for example, teaching material on “YouTube”; External Tool for giving assignments on the construction of BLOGS on padlet.com and WIKI to create Wikis for collaborative learning.

The feature of the workshop was also utilized by assigning subtopics to the students. Dates and time for the opening and closing of the workshop were displayed on the “Announcement” section and communicated through WhatsApp Messenger. All students were notified by the “Announcements” posted on the Dashboard before the commencement of the workshop on a particular topic. The workshop was utilized as a formative peer-assessment tool for blogs and Wikis created. Access to all assignments on Blogs and Wikis were time-restricted for a week since their announcement.

A quiz was another tool utilized for formative assessment in the form of Scenario-based One-Best Choice Questions. Two Quizzes were Conducted during the 16 weeks and were time-bound. The results with detailed statistics of workshop and quiz were available for display on announcement section on Dashboard, immediately after the set deadline was over. Awards consisted of Virtual Badges created by the teachers using external tools given to the first three scorers as Gold, Silver and Bronze. They were made available for display on Dashboard as an incentive to excel.

The face-to-face component of blended learning

The face-to-face component of blended learning comprised of history taking, clinical examination of patients in out-patients departments, emergency room and perioperative management of surgical patients in the ward, operation theatre and Intensive Care Unit. This included operations on weekly emergency days and twice on elective days for limited tumour surgeries. In the Operating Room, strict Standard Operating Procedures (SOPs) were followed under the supervision of faculty and consultants. Whereas in the ward, bedside teaching rounds were done daily by a consultant and two postgraduates to maintain social distancing and reduction of exposure.

Study design and participants

Our study was designed as a prospective single-centre online survey conducted at the Department of Surgery, Civil Hospital Karachi. Approval from the Institute Review Board (IRB) of Dow University of Health Sciences (DUHS) was obtained. A total of 33 General Surgical trainees were enrolled in the teaching course designed on Moodle LMS.

Study tool

The completion of the course spanned over four months, from May 2020 till August 2020. The approval from IRB was sought, and soon after obtaining it a questionnaire was designed online in March 2021, and all the participants were encouraged to fill it out. The participation remained voluntary and anonymous. The questionnaire was divided into two parts. The first consisted of questions about demographics. The second part was feedback on blended teaching & learning course for postgraduate surgical trainees, particularly regarding their effectiveness, accessibility, and satisfaction. The response was themed on a Likert scale with 1 representing ‘strongly disagree’; 2 ‘disagree’; 3 ‘uncertain’; 4 ‘agree’; 5 ‘strongly agree’. The questionnaire was based on a similar well-cited study [13].

Statistical analysis

All the collected data were analyzed in SPSS 23. Categorical variables such as gender and hierarchy of residents were presented as percentages and frequency. The analyses included descriptive statistics and a chi-square test. The level of statistical significance was taken as 0.05.

## Results

Demographics

The 14-item questionnaire was administered to the postgraduate trainees of the Surgical Unit in Civil Hospital Karachi. A total of 33 postgraduate trainees participated in the study, all volunteered to participate in the study and the response rate was 100%. Nearly three-fourths of the trainees were females (75.8%) which reflects the majority of the trainees in our surgical unit. Year 1 trainees constituted nearly a third (33.3%) of the responders, followed by Year 2, 3 and 4 as listed in table 1.

**Table 1:** Demographic characteristics of the postgraduates N: Number of participants

VARIABLES	CLASSIFICATION	FREQUENCY (N=33)	PERCENTAGE (%)
Age ( in years)	25	4	12.1
	26	9	27.3
	27	8	24.2
	28	9	27.3
	29	3	9.1
Gender	Male	8	24.2
	Female	25	75.8
Year	Year 1	11	33.3
	Year 2	10	30.3
	Year 3	7	21.2
	Year 4	5	15.2

Postgraduate trainees' response in regards to the effectiveness of Moodle LMS- as the online component of blended learning

The trainees had a positive response towards Moodle. More than half of the participants agreed that Moodle LMS was user-friendly, provided a collaborative environment (54.5%), and was compatible with common browsers and hardware (57.6%). It was easy too, but it also provided various learning resources and multimedia tools to create attractive activities making education easy for learners. Almost two-thirds of the participants (69.7%) believed that Moodle provided an environment conducive to active class engagements. 51.5% of students were of the consensus that the results from the formative assessment were helpful and immediately available. This has led to their increased preference for online scenario-based quizzes (69.7%).

Postgraduate trainees' perception towards blended learning

Regarding the utility of blended learning in surgery, the postgraduates found it conducive to understanding common surgical problems as projected by a percentage of 45.4%. Most postgraduates' online and face-to-face component of blended learning was found to be useful in acquiring surgical knowledge and skills (39.4% & 42.4%, respectively). However, when it came to sophisticated and advanced aspects of learning in surgery, such as interpretation of laboratory and clinical investigations and the case-based learning and post-emergency meeting, the trainees were unsure about the usefulness of blended learning. This owes to the fact that on-field learning is perhaps more essential in practical learning. Nevertheless, nearly half of the trainees (54.5%) would recommend blended learning education in surgical training programs. The response of the participants themed on the Likert scale are illustrated in table 2.

**Table 2:** Postgraduates’ attitude towards blended learning LMS: Learning Management System; PC: Personal Computer; SD: Standard Deviation.

SR No	Questions	Strongly disagree	Disagree	Uncertain	Agree	Strongly agree	Mean ±SD
1	Moodle LMS was user friendly and provided customizable learning platform.	5 (15.2%)	1 (3%)	9 (27.3%)	18 (54.5%)	0	3.21±1.08
2	Moodle LMS is compatible with common browsers on common hardware (PCs, mobile devices, tablets etc.)	3 (9.1%)	1 (3%)	9 (27.3%)	19 (57.6%)	1 (3%)	3.42±0.969
3	Moodle LMS provides and facilitates a collaborative environment among students.	3 (9.1%)	2 (6.1%)	8 (24.2%)	18 (54.5)	2 (6.1%)	3.42±1.03
4	Moodle is a good online learning platform as variety of resources and tools are available	3 (9.1%)	4 (12.1%)	5 (15.2%)	16 (48.5%)	5 (15.2%)	3.48±1.17
5	Moodle provides a learning environment for active class engagement (virtual & non-virtual)	3 (9.1%)	3 (9.1%)	4 (12.1%)	23 (69.7%)	0	3.42±1.00
6	The results and statistics of Formative assessment are immediately available after the test is over on Moodle LMS	4 (12.1%)	1 (3%)	8 (24.2%)	17 (51.5%)	3 (9.1%)	3.42±1.12
7	The breakdown of results of formative assessment on Moodle LMS is helpful to evaluate my learning performance.	3 (9.1%)	1 (3.0%)	5 (15.2%)	21 (63.6%)	3 (9.1%)	3.60±1.02
8	The comprehensive learning activities in the blended learning program has enhanced my understanding of common surgical problems.	3 (9.1%)	5 (15.2%)	10 (30.3%)	15 (45.5%)	0	3.12±0.99
9	I would prefer more surgical scenario-based online content and online quizzes.	4 (12.1%)	0	0	6 (18.2%)	23 (69.7%)	4.33±1.31
10	The online component of the blended program helped me in acquiring and understanding surgical knowledge except for surgical skills.	4 (12.1%)	7 (21.2%)	5 (15.2%)	13 (39.4%)	4 (12.1%)	3.18±1.26
11	Blended learning has helped me to interpret common laboratory and radiological investigations	3 (9.1%)	1 (3%)	20 (60.6%)	9 (27.3%)	0	3.06±0.82
12	The face-to-face component of Blended Learning helped me develop specific clinical and surgical skills	3 (9.1%)	5 (15.2%)	6 (18.2%)	14 (42.4%)	5 (15.2%)	3.39±1.19
13	Case-based learning and post-emergency discussions on ZOOM meetings helped me in applying knowledge for managing common surgical pathologies and surgical emergencies.	3 (9.1%)	0	2 (6.1%)	19 (57.6%)	9 (27.3%)	3.93±1.08
14	I would recommend a blended learning program/course for surgical training to the rest of my colleagues	5 (15.2%)	1 (3%)	1 (3%)	18 (54.5%)	8 (24.2%)	3.69±1.31

For the postgraduate trainees, the blended learning program in general and the face-to-face component, in particular, was useful in enhancing their surgical and developing skills and linked statistically to their year of training, as depicted in table 3.

**Table 3:** Correlation between the preferences of the postgraduate trainees towards blended learning and their year of residency LMS: Learning Management System; PC: Personal Computer

SR No	Questions	Academic Year	Strongly disagree	Disagree	Uncertain	Agree	Strongly agree	P-value
1	Moodle LMS was user friendly and provided a customizable learning platform.	Year 1	1	1	5	4	0	0.185
		Year 2	2	0	0	8	0	
		Year 3	2	0	1	4	0	
		Year 4	0	0	3	2	0	
2	Moodle LMS is compatible with common browsers on common hardware (PCs, mobile devices, tablets etc.)	Year 1	1	0	2	7	1	0.495
		Year 2	0	1	2	7	0	
		Year 3	2	0	3	2	0	
		Year 4	0	0	2	3	0	
3	Moodle LMS provides and facilitates a collaborative environment among students.	Year 1	1	0	3	7	0	0.309
		Year 2	2	0	3	4	1	
		Year 3	0	2	1	4	0	
		Year 4	0	0	1	3	1	
4	Moodle LMS is a good online learning platform as a variety of resources and tools are available	Year 1	1	2	1	5	2	0.418
		Year 2	2	0	3	4	1	
		Year 3	0	2	1	4	0	
		Year 4	0	0	0	3	2	
5	Moodle provides a learning environment for active class engagement (virtual & non-virtual)	Year 1	1	1	3	6	0	0.257
		Year 2	2	0	1	7	0	
		Year 3	0	2	0	5	0	
		Year 4	0	0	0	5	0	
6	The results and statistics of Formative assessment are immediately available after the test is over on Moodle LMS	Year 1	0	0	5	6	0	0.092
		Year 2	2	1	0	4	3	
		Year 3	2	0	1	4	0	
		Year 4	0	0	2	3	0	
7	The breakdown of results of formative assessment on Moodle LMS are helpful to evaluate my learning performance.	Year 1	1	1	0	9	0	0.102
		Year 2	2	0	1	4	3	
		Year 3	0	0	3	4	0	
		Year 4	0	0	1	4	0	
8	The comprehensive learning activities in the blended learning program has enhanced understanding of common surgical problems	Year 1	1	2	2	6	0	0.003
		Year 2	2	3	0	5	0	
		Year 3	0	0	7	0	0	
		Year 4	0	0	1	4	0	
9	I would prefer more surgical scenario based online content and online quizzes.	Year 1	1	0	0	2	8	0.300
		Year 2	1	0	0	0	9	
		Year 3	2	0	0	2	3	
		Year 4	0	0	0	2	3	
10	The online component of blended program helped me in acquiring and understanding surgical knowledge except surgical skills.	Year 1	1	2	2	3	3	0.611
		Year 2	2	2	1	5	0	
		Year 3	1	3	1	2	0	
		Year 4	0	0	1	3	1	
11	Blended learning has helped me to interpret common laboratory and radiological investigations	Year 1	1	1	5	4	0	0.592
		Year 2	2	0	7	1	0	
		Year 3	0	0	4	3	0	
		Year 4	0	0	4	1	0	
12	The face-to-face component of blended Learning helped me develop specific clinical and surgical skills	Year 1	1	1	1	5	3	0.004
		Year 2	2	4	0	4	0	
		Year 3	0	0	5	2	0	
		Year 4	0	0	0	3	2	
13	Case based learning and post emergency discussions on ZOOM meetings helped me in applying knowledge for managing common surgical pathologies and surgical emergencies.	Year 1	1	0	0	7	3	0.095
		Year 2	0	0	2	3	5	
		Year 3	2	0	0	5	0	
		Year 4	0	0	0	4	1	
14	I would recommend blended learning program/course for surgical training program to rest of my colleagues	Year 1	1	0	1	4	5	0.543
		Year 2	2	1	0	6	1	
		Year 3	2	0	0	4	1	
		Year 4	0	0	0	4	1	

## Discussion

In March 2020, there was a sudden interruption observed in medical education due to the COVID-19 pandemic causing a dramatic increase in hospitalizations and mortality worldwide. It was creating a gap in teaching and learning process that was required to be filled. Globally, most medical institutions, including those in Pakistan, shifted the mode of teaching to e-learning. To enhance the learning experience, in particular for post graduate training, blended learning was adopted, which led to innovation in many aspects of medical education [14]. There is an integration of an online, both synchronous and asynchronous, with the conventional face-to-face learning experience in blended learning. Its online component has many advantages, especially being accessible anytime and at any place, giving students room to learn at their own pace, and with a potential of increasing interaction between teachers and students [15].

In our study, we tried to look at our trainees' opinions toward major challenges that they faced during their new experience of the blended learning system, particularly its online component, i.e., Moodle LMS, its benefits, limitations, and overall satisfaction future perspectives.

Trainees' perception and acceptance have been a satisfactory and positive response towards the blended learning system. It was received favourably by 54.6 % in our study, similar to previous studies conducted on blended learning in the past [16-20]. In a study published in the American Journal of Surgery, the response of most surgical residents was positive in regards that the approach of blended learning was providing a curriculum that educates them virtually but face-to-face in person. However, the majority of their attendings did not agree. The difference in perception of the attendings or supervisors is probably due to the generation gap and wider exposure and application of technology by residents who have grown up in this technological age. While residents find it easier to grasp the web-based platforms and various online tools, our teachers may not be as familiar or have expertise in applying them for online teaching. Covid-19 pandemic has provided an opportunity for development in virtual learning for students, particularly in instructional & learning strategies for faculty & teachers [17].

In a study in Scotland, most postgraduate orthopaedic trainees (96%) were satisfied with the quality and relevance of virtual teaching. They stated that online teaching should continue to be a part of the delivery of postgraduate training [20]. However, a study in Malaysia reported a mixed review of blended learning by educators and trainees. Though blended learning was perceived to encourage student-teacher interactions, it was feared that adapting to it remained a challenge for educators and learners and may cause an extra burden to them [21].

The preference of the blended learning approach was influenced by the year of training, as seen in our study. This can be attributed to different factors based on hierarchy. To our surprise, similar statistics were also seen in the review done by Amir et al. on Indonesian dental surgery students [13]. The year one resident, being the most junior, particularly found this helpful in terms of the leniency of working hours and on-call duties. While on the other hand, year four residents preparing for the exit exam found extra hours beneficial for exam preparation and research assignments. This again is perhaps in context with studies that showed that the pandemic had blessed trainees with reduced working hours, rendering them less likely to burnout [22].

Surgery being a work-based, hands-on training curriculum, requires the residents to attend elective and emergency theatres, outpatient clinics and perform emergency duties. Hence, when it comes to obtaining the clinical history, performing a clinical examination and acquiring surgical skills, the face-to-face component was preferred by our trainees as compared to its online component. The pandemic has adversely affected their on-table training as most programs, including ours, have become compromised and restricted to follow SOPs and maintain social distance [13,16,22]. However, when asked about the interpretation of laboratory and radiological investigations, case-based learning and post-emergency meetings, our trainees were unsure about the usefulness of blended learning. This borderline scoring of quality indices compared to a traditional class may be secondary to decreased physical interaction. Despite the ease of availability of teachers, via either online or phone, there was a lack of in-person guidance and instructions. In a study in Malaysia, some trainees found it difficult to adapt to these changes in such a short span. The students felt uncomfortable taking examinations online due to the possibility of cheating. Many of the trainees felt their training and knowledge were incomplete due to a lack of formal training before the COVID-19 era. Despite these limitations and challenges, the results showed increased positivity among the students for blended learning during the pandemic. That online learning leads to better student participation, interaction & collaboration [23].

As far as comprehensive learning activities in the blended learning program were concerned, many of our residents agreed that they enhanced their understanding of common surgical problems. This is perhaps secondary to the increased utilization of gadgets for Virtual Classroom meetings and digital learning materials. Also, this is supported by the study conducted by Bridges SM, that utilization of e-learning technology with conventional teaching has led to better understanding and engagement of students with their studies [24]. We cannot ignore that trainees could communicate better with their teachers or peers by just texting or calling them in times of query. The interaction was made even better during online team meetings. Thus, a shy or introverted learner gets to boost his or her learning as well [23], as affirmed by our year 1, Year 2 residents followed by year 4.

Regarding the online component of our study, i.e., Moodle LMS, it was found not only to be user-friendly but also to provide a platform for students to enhance their cognitive knowledge in a collaborative environment. It helps teachers give assignments using its tool such as assignments, implement web-based peer assessment and take formative assessments using a tool of quiz [10,21]. Implementation of distance e-learning, especially in a country where a large portion of our population lives under poverty, can be challenging [25]. However, our postgraduate trainees felt that Moodle LMS was very browser and hardware friendly and provided them with the accessibility to finish assignments at their own pace, thus remarkably utilizing the "work-from-home" policy.

Moodle has provided an environment for active class engagements for trainees with their teachers, which is necessary to lay a deep connection with the material beyond just mere reading and to listen and helps in the clinical application of the information learnt and developing skills [23].

Blended learning, using Moodle LMS, is an effective combination of using various online tools in an interactive learning environment. The Moodle LMS has multiple built-in tools for assignments and exercises such as wikis. This workshop can improve student's ability to understand and analyze the topics of the course critically. It has an online assessment tool, such as quizzes in the form of multiple-choice questions, that can be answered anywhere without being face-to-face. The learning processes using Moodle LMS make the students motivated for interactive and collaborated learning, asynchronously and synchronously, by incorporating ZOOM Meeting assisted by WhatsApp [26].

Our postgraduate trainees were engaged in active online learning on Moodle LMS, utilizing different means of communication such as discussions forum for asynchronous and chat (WhatsApp) for synchronous communication, which boosted peer to peer and peer to instructor relationships. The online ZOOM meeting tool was incorporated with our Moodle LMS and was used for interactive synchronous sessions of post-emergency surgical Meetings twice weekly.

The greatest asset of Moodle projected in our survey was the Quiz tool. It has gained the best outcome as the results were made immediately available for students to see and assess their level of knowledge. The online examination allowed the assessment to be done at the candidates' comfort zone. The main aim of these quizzes was not only personal evaluation but also learning. The "open book" type nature of these quizzes allowed them to learn those topics that they have not gone through before. This is why many of the participants preferred to have more online quizzes.

Many limitations certainly need further consideration. Our study has a limited sample size and represents the view of postgraduate surgical trainees of our hospital only. The clinical significance of our study will increase further if it includes trainees from other hospitals and cities too. Our study is a mere reflection of postgraduate trainees' perception regarding the LMS. However, it does not measure its effect on their level of performance, which will be necessary if changes are to be made in transforming the curriculum. The applicability and acceptability of the LMS may increase further if the duration of the study is also increased. Despite these limitations, there is an increased positivity among the students about blended learning during the pandemic.

## Conclusions

Moodle LMS as the online part of blended learning is beneficial in postgraduate training in surgery in a COVID-19 pandemic situation. However, the face-to-face component of blended learning is still an essential component for the learning process for a surgical trainee. This is particularly for acquiring psychomotor skills such as examining a patient and acquiring operative surgical skills in a work-based learning setup. Using a combination of conventional face-to-face and virtual instruction, blended learning has a strong potential to stay in the future for surgical postgraduate training. Not many studies have been done previously on surgical post-graduate trainees doing blended learning programs. Therefore we recommend further utilization of this modality in future educational aspects.

## Appendices

Questionnaire

Section A: Demographics

Age:

Gender:

Year of residency:

Section B: Feedback on blended learning

**Table 4:** Feedback on blended learning themed on Likert scale LMS: Learning Management System; PC: Personal computer

SR No	Questions	Strongly disagree	Disagree	Uncertain	Agree	Strongly agree
1	Moodle LMS was user friendly and provided customizable learning platform					
2	Moodle LMS was compatible with common browsers on common hardware (PCs, mobile devices, tablets etc.)					
3	Moodle LMS provided and facilitated a collaborative environment among students					
4	Moodle was a good online learning platform as a variety of resources and tools were available					
5	Moodle provided a learning environment for active class engagement (virtual & non-virtual)					
6	The results and statistics of formative assessment were immediately available after the test was over on Moodle LMS					
7	The breakdown of results of formative assessment on Moodle LMS were helpful to evaluate my learning performance					
8	The comprehensive learning activities in the blended learning program has enhanced understanding of common surgical problems					
9	I would prefer more surgical scenario based online content and online quizzes					
10	The Online component of blended program helped me in acquiring and understanding surgical knowledge except surgical skills					
11	Blended learning has helped me interpret common laboratory and radiological investigations					
12	The face-to-face component of blended learning helped me develop specific clinical and surgical skills					
13	Case based learning and Post emergency discussions on ZOOM meetings helped me in applying knowledge for managing common surgical pathologies and surgical emergencies					
14	I would recommend blended learning program/course for education in surgical training program to my colleagues					
